# Healthcare worker views on antimicrobial resistance in chronic respiratory disease

**DOI:** 10.1186/s13756-025-01515-8

**Published:** 2025-01-22

**Authors:** Sachin Ananth, Adekunle O. Adeoti, Animesh Ray, Peter G. Middleton, Miquel Ekkelenkamp, Stephanie Thee, Anand Shah, the AMR-Lung Clinical Research Collaboration, Abayomi Fadeyi, Abdoul Risgou Ouedraogo, Addishiwot Melesse Seminew, Adele Roux, Adnan Zafar, Aizhamal Tabyshova, Aleksandra Barac, Alex Gileles-Hillel, Alexander Kiefer, Alexandra Hebestreit, Alice Tebboth, Amelia Shoemark, Ana Machado, André Santos-Silva, Andrea Gramegna, Andriy Serediuk, Angela Tramontano, Anna Salina, Annie Navarro Rolon, Anoop Prakash, António Gonçalves, Aran Singanayagam, Arun H. Mahadevaiah, Asha Muthusami, Avinash Aujayeb, Ayşe Önal Aral, Barbara Kahl, Ben Huggon, Bohdana Pereviznyk, Braulio Chevalier Vidal, Bukar Bakki, Bulent Karadag, Bushra Ahmed, Calmés Doriane, Cao Pham Ha Giang, Carmelo Sofia, Catia Cilloniz, Cátia Paixão, Charl Verwey, Charles Feldman, Charlotte Carter, Chiara Premuda, Chizoba Efobi, Clementine Fraser, Corentine Alauzet, Damir Vukoja, Danial Naqvi, Daniela Maria Cirillo, Dareen Marghlani, Daryl Butler, David Abelson, David Stickells, Deepa Kumari Shrestha, Deepa Patel, Devesh J. Dhasmana, Devi Jyoti Dash, Diana Ergle, Dilara Ömer Topçu, Dominic L. Sykes, Dorina Rama Esendagli, Dumitras Tatiana, Efthymia Papadopoulou, Elsa Branco, Eva Van Braeckel, Evans Frexon Liseki, Evie Alexandra Robson, Fapohunda Temitope Victoria, Maria de Fatima Magalhaes Gonzaga, Felix Bongomin, Felix C. Ringshausen, Felix Manyeruke, Freddy Frost, Friso de Weert, Garante Carmela Melania, Garry McDonald, Geneviève Héry-Arnaud, Giancarlo De Leo, Gina Amanda, Gioia Piatti, Giovanna Manfredini, Grillon Antoine, Guillaume Thouvenin, Gunar Günther, Hélida Conceição Cavalcante Torres, Helle Krogh Johansen, Henny Azmanov, Hussein Elkhayat, Hussein Mohamed Ahmed, Ian Clifton, Ignacio Martin-Loeches, Indiane Putri Ningtias, Ines Azevedo, Inge Muylle, Irfan Shafiq, Iwein Gyselinck, Joel Israëls, Jaber S. Alqahtani, James Ayodele Ogunmodede, Jamil Jubrail, Jatin G. Nagar, Jeanne-Marie Perotin, Jimstan Periselneris, Jo Congleton, Johnmary T. Arinze, Joseph Fadare, Joy Eze, Justus Simba, Kartik Kumar, Katharine Hurt, Kay Roy, Koen Verbeke, Kristi Reveli, Krystyna Poplawska, Kwok Wang Chun, Lawani Olufemi Ademola, Leidy Prada, Leonardo Gori, Letizia Corinna Morlacchi, Linda Aprillia Rolobessy, Lisa Nwankwo, Lorenzo Carriera, Loskova Elena Vladimirovna, Lydia Finney, Mai S. Elsheikh, Malvina Hoxha, Marcos I. Restrepo, Margarete Lopes Teixeira Arrais, Maria Gabrovska, Maria Grazia Cagnazzo, Maria Joana Catarata, Marialuisa Bocchino, Mario Di Stasio, Marrah Lachowicz-Scroggins, Mary Wambura, Matlawene John Mpe, Matthew Pavitt, Mattia Nigro, Melanie Sue Collins, Michelle Uno, Miguel Gallego, Milind Sathe, Mine Kalyoncu, Mohammad Abdullah, Mona Lichtblau, Mukesh Singh, Mwanaada Ahmad Kilima, Natalie Lorent, Nazanin Farahbakhsh, Ngoc Duong-Minh, Nguyen Pham Anh Hong, Nicola Ronan, Nicola Travaglini, Nilotpal Bhattacherjee, Nilüfer Aylin Acet Öztürk, Nina Ratu Nur Kharima, Niranjan Chandramal Lehupe Bandarage, Nishith Kumar, Nita Corry Agustine Nias, Nwosu Nnamdi Ikechukwu, Oleksandr Mazulov, Olga Bielousova, Olga Mashedi, Omer Elneima, Ophir Bar-On, Özge Aydın Güçlü, Pabitra Banerjee, Pavel Yordanov, Pedro Gonçalo Ferreira, Pieter Goeminne, Prakash Mohan Jeena, Priti Kenia, Priyanka Poda, Pujan H. Patel, Rafiuk Cosmos Yakubu, Rameesha Khalid, Ranganath Thippanahalli Ganga, Rasheedat Mobolaji Ibraheem, Ravini Karunatillake, Rawya Ahmed, Ricardo Figueiredo, Richard Hewitt, Ridzuan Mohsin, Rodrigo Abensur Athanazio, Rohit Kumar, Rosanel Amaro, SRaghul Raj, Sabi Hippolyte, Sabrine Louhaichi, Salvatore Tripodi, Sandra Rovira-Amigo, Sanem Eryılmaz Polat, Sara Manti, Sarah Loof, Saurabh Singh, Sega Pathmanathan, Serena Romeo, Shirley V. Cuan-Escobar, Silvia Castillo-Corullón, Sinchuk Nataliya, Siobhan B. Carr, Siyu Dai, Snezhina Lazova, Sonja van Scheijen, Sophie Gohy, Soumitra Mondal, Srimali Wijesundara, Stavros Tryfon, Stefano Aliberti, Stephan Illing, Suleiman Sherifat Tinuke, Sumudu Withanage, Susanne Hämmerling, Tariq Qadeer, Tavs Qvist, Tehreem Ahmad, Temitope Victoria Fapohunda, Thomas Guillard, Till Othmer, Tim Felton, Tony De Soyza, Toufic Chaaban, Vanessa Kahr, Vânia Fernandes, Vera Clérigo, Veroniek Saegeman, Vikram Damaraju, Vipula Rasanga Bataduwaarachchi, Vivek Gundappa, Yannick Vande Weygaerde

**Affiliations:** 1 London North West University Healthcare NHS Trust, London, UK; 2 Ekiti State University Teaching Hospital, Ado Ekiti, Nigeria; 3 All India Institute of Medical Sciences, New Delhi, India; 4 CITRICA, Department of Respiratory and Sleep Medicine, Westmead Clinical School, University of Sydney, Sydney, Australia; 5 University Medical Center Utrecht, Utrecht, Netherlands; 6 Charité-Universitätsmedizin Berlin, Berlin, Germany; 7 Berlin Institute of Health (BIH), Berlin, Germany; 8 Royal Brompton Hospital, Guy’s and St. Thomas’ NHS Foundation Trust, London, UK; 9 MRC Centre of Global Infectious Disease Analysis, Imperial College London, London, UK

**Keywords:** Infection and inflammation, Infection control, Respiratory infections, Tuberculosis, Chronic bronchiectasis, Clinical epidemiology, Clinical respiratory disease

## Abstract

**Background and objective:**

Antimicrobial resistance (AMR) is a global crisis, however, relatively little is known regarding its impact in chronic respiratory disease and the specific challenges faced by healthcare workers across the world in this field. We aimed to assess global healthcare worker views on the challenges they face regarding AMR in chronic respiratory disease.

**Methods:**

An online survey was sent to healthcare workers globally working in chronic respiratory disease through a European Respiratory Society clinical research collaboration (AMR-Lung) focussed on AMR in chronic lung disease. Responses from different geographic regions were analysed.

**Results:**

279 responses were received across 60 countries. 54.5% of respondents encountered AMR in chronic respiratory disease weekly. There were differences in perceived high-priority diseases and species with AMR burden between Europe, Asia and Africa. 76.4% of respondents thought that inappropriate antimicrobial prescribing in chronic respiratory disease was common. However, only 43.4% of respondents thought that there were adequate antimicrobial stewardship programmes in their area for chronic respiratory disease, with limited availability in outpatient (29.0%) and ambulatory settings (24.7%). Developing rapid diagnostics for antimicrobial susceptibility (59.5%) was perceived to be the most common challenge in implementing antimicrobial stewardship, with an improved understanding of regional epidemiology of AMR strains the most important factor to improve outcome (55.2%).

**Conclusions:**

AMR has significant perceived burden in chronic respiratory disease by healthcare professionals globally. However, current implementation of antimicrobial stewardship is limited, with significant challenges related to the availability of rapid diagnostics and understanding of regional epidemiology of AMR strains.

**Supplementary Information:**

The online version contains supplementary material available at 10.1186/s13756-025-01515-8.

## Background

Antimicrobial resistance (AMR) is one of the top global public health and development threats, with estimated direct responsibility for 1.3 million deaths in 2019 and projections indicating an increase to 10 million deaths by 2050 [1, 2]. AMR has complex drivers, including the improper use of antimicrobials in agricultural practices, veterinary medicine and human healthcare [3]. This contributes significantly to healthcare expenses and is predicted by the World Bank to lead to an extra $1 trillion in healthcare expenditures by 2050 [4].

Antimicrobial therapy plays a critical role in the management of patients with chronic respiratory disease (including chronic obstructive pulmonary disease, bronchiectasis and asthma), which is a major contributor to the global healthcare burden with an estimated prevalence of 454.6 million cases, and the third leading cause of death (4 million cases annually) [5]. Although recent studies have detailed the presence of AMR within chronic respiratory disease and associated AMR with poor clinical outcome, there is limited data on global AMR prevalence in chronic respiratory disease, with a lack of antimicrobial stewardship policy within the field [6–8]. Individuals with chronic respiratory diseases often require recurrent or prophylactic antimicrobial therapies to treat or prevent exacerbations, emphasising the importance of understanding microbial epidemiology alongside the need for tailored treatment strategies and antimicrobial stewardship programmes [9]. For this reason, the AntiMicrobial Resistance in Lung infections (AMR-Lung) clinical research collaboration was established by the European Respiratory Society (ERS) to better understand the impact of AMR in chronic respiratory disease, the role of current and novel diagnostic techniques, and to refine therapeutic approaches and antimicrobial stewardship policy for AMR-related respiratory infections [10].

Healthcare workers play a critical role in appropriate antimicrobial prescription, patient education on antimicrobial use and the implementation of antimicrobial stewardship programmes. Hence, understanding healthcare workers’ perspectives on AMR in chronic respiratory disease is critical to developing and implementing effective strategies to combat this rapidly growing global threat. However, practices and policies vary considerably globally, and although prior surveys have highlighted general AMR perception amongst healthcare practitioners, there is little understanding of healthcare worker exposure, understanding and practice relating to AMR within chronic respiratory disease [11–13]. To address these gaps, we conducted a global survey among healthcare professionals involved in the care of patients with chronic respiratory disease. The objectives of the study were to explore healthcare workers' views and experiences regarding AMR in chronic respiratory disease management and policy, and to assess perception about antimicrobial stewardship principles.

## Methods

### Survey design

An online survey was created, covering the main themes of AMR and antimicrobial stewardship in chronic respiratory disease (Table S1). Survey questionnaire items were adapted from previous surveys of healthcare workers on AMR, with further items developed through steering group consensus comprising AMR experts from within the AMR-Lung clinical research collaboration [13–16].

The survey comprised four sections: (1) Respondent demographics; (2) Importance of AMR in chronic respiratory disease; (3) Antimicrobial stewardship and (4) Research priorities. The first section focussed on the respondents’ demographic details and included items to understand respondents’ level of experience, work setting and which respiratory conditions they primarily manage. The second section covered the importance of AMR in respiratory disease, including the impact of AMR on respondents’ clinical practice, which resistant species were considered most important, and priorities for improving management of AMR in chronic respiratory disease. The third section included items regarding the content and quality of antimicrobial stewardship programmes in the respondents’ region of practice. The last section included items detailing respondents’ views on the priorities for future research in AMR in chronic respiratory disease.

Items either used a 5-point Likert scale (strongly disagree to strongly agree) or included a limited number of options to highlight the most important priorities. Free-text boxes were provided as an option in some items in case respondents felt that an important answer was not included in the list of provided options. For items regarding specific antimicrobial resistant species, the species chosen were based on the most common resistant species found in respiratory disease globally [1]. The online survey was created using the Google Forms survey administration software (Google, California, USA).

### Data collection

Survey distribution occurred over a 6-week period between 2nd February to 18th March 2024, and included members of the ERS AMR-Lung clinical research collaboration, alongside respiratory clinicians and scientists via newsletters of national respiratory societies, including the British Thoracic Society and Pan Africa Thoracic Society.

### Statistical analysis

Responses were collated and statistical analysis performed using GraphPad Prism 10.2, with continuous data presented as mean ± standard deviation unless stated otherwise. Geographical sub-group analysis was performed for the continents of Asia, Africa and Europe. Insufficient responses were available for the other major continents: Australasia (3 responses), North America (5 responses) and South America (7 responses) to be included in sub-group analysis. Chi-squared test was used to compare categorical data between the three continents analysed. For between-continent differences, the significance level α was set as 0.016 (0.05 divided by 3) to account for multiple comparisons.

## Results

**Fig. 1:**
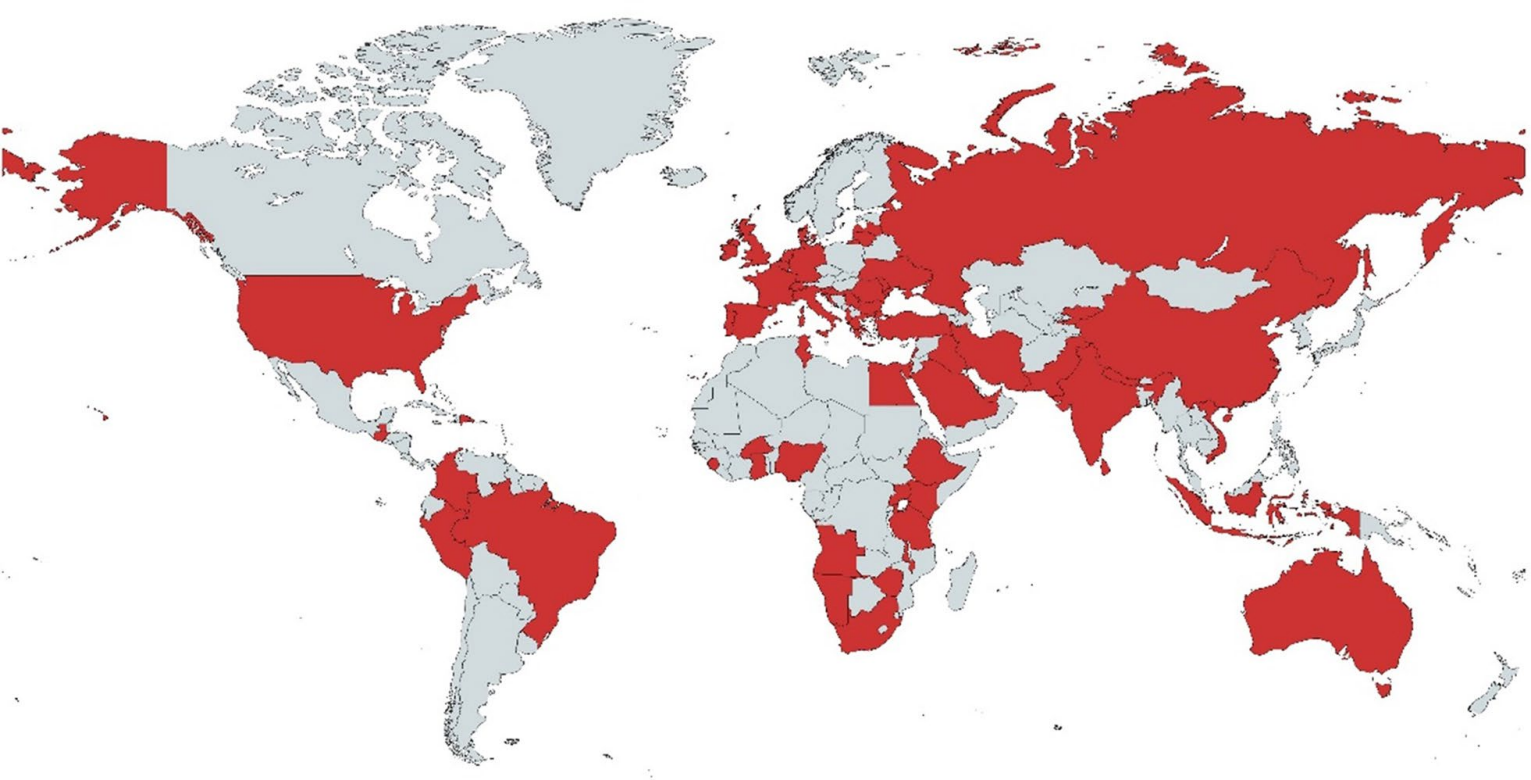
World map showing the survey respondents’ countries of origin (highlighted in red). Map created using MapChart.net [39]

279 responses were received from 60 countries across 6 continents (Fig. 1), with the majority from Europe (159/279; 57.0%) followed by Asia (63/279; 22.6%) and Africa (42/279; 15.1%). Respondents were practising healthcare workers for 15.8 ± 9.0 years, with 91.4% (255/279) of respondents working as clinicians (Table S2 shows the occupations of the respondents). Figure S2 shows further demographic details of the respondents, with 65.2% (182/279) working in tertiary or quaternary care, and the majority managing adult patients or both adult and paediatric patients, with just 15.1% (42/279) managing paediatric patients only. 70.6% (197/279) of respondents prescribed and/or reviewed antimicrobial prescriptions daily.

**Fig. 2:**
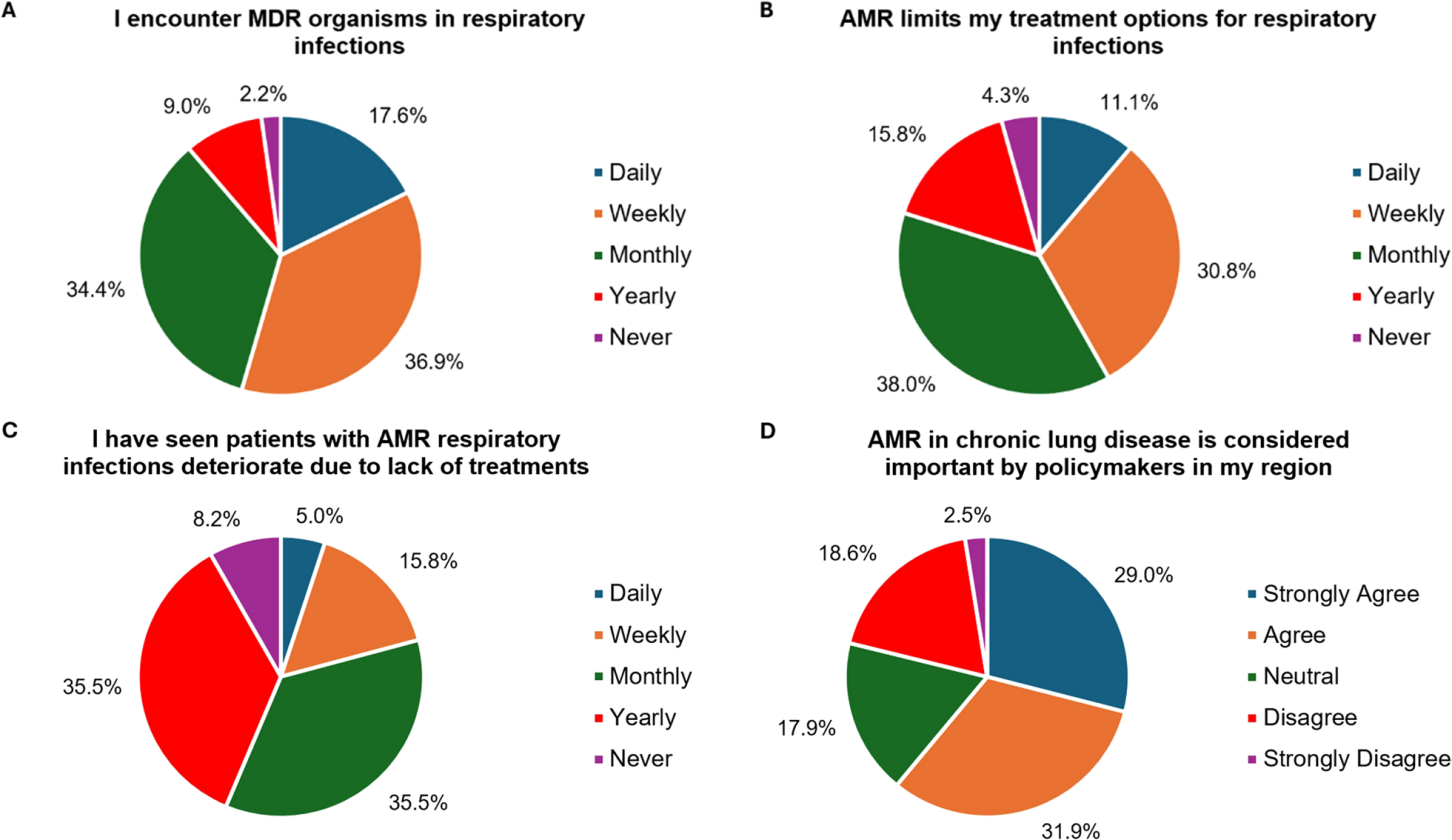
The burden of antimicrobial resistance (AMR) in chronic respiratory disease. **A** Frequency that respondents encounter multi-drug resistant (MDR) organisms in respiratory infections. **B** Frequency that respondents’ treatment options are limited due to AMR in respiratory infections. **C** Frequency that respondents see patients with respiratory infections clinically deteriorate due to a lack of treatment options as a result of AMR. **D** The extent to which respondents feel that policymakers in their regions consider AMR in chronic lung disease to be an important topic

54.5% (146/279) of respondents encountered multi-drug resistant (MDR) organisms in respiratory infections daily or weekly, with 41.9% (114/279) stating that AMR limited their treatment options for respiratory infections daily or weekly (Fig. 2A, B). 20.8% (58/279) of respondents had seen patients with respiratory infections clinically deteriorate due to a lack of treatment options because of AMR daily or weekly (Fig. 2C), with 10.1% (28/279) of respondents having seen patients die daily or weekly due to AMR (Figure S3). 60.9% (170/279) of respondents felt that AMR in chronic respiratory disease was considered an important topic by policymakers in their region (Fig. 2D). There were no differences in the perception of AMR based on the respondents’ continent of practice.

**Fig. 3:**
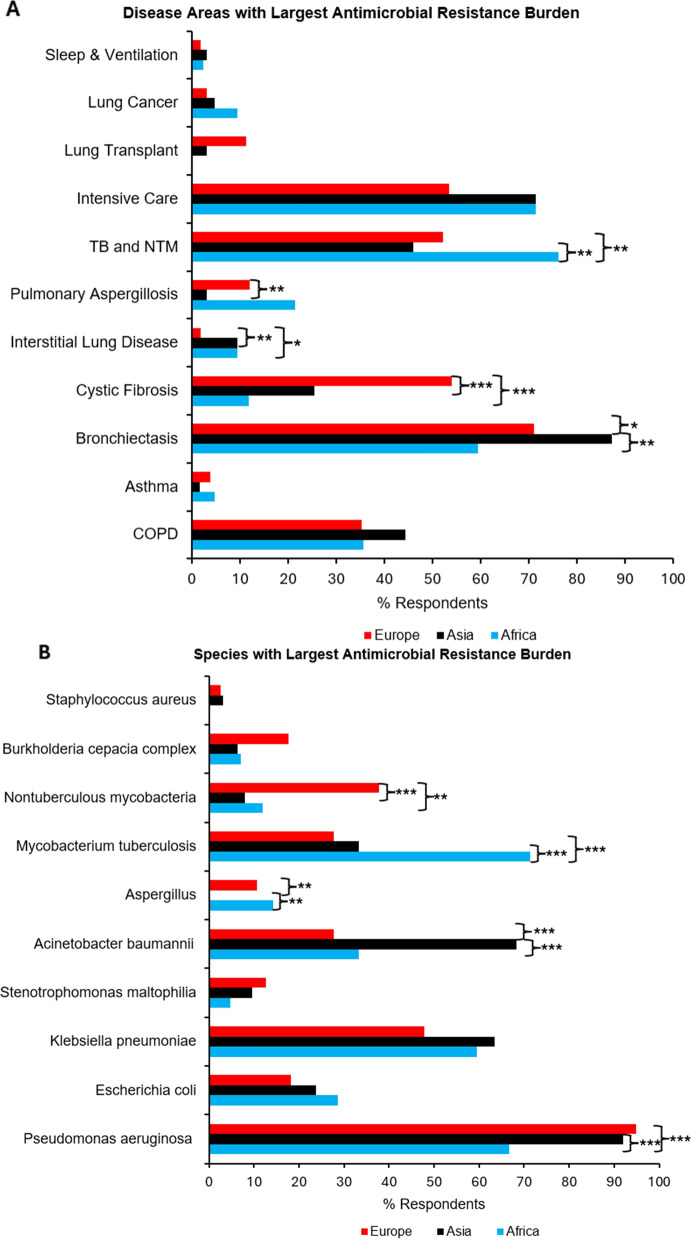
Geographical differences in the perceived burden of antimicrobial disease. **A** Disease areas with the greatest perceived burden of antimicrobial resistance. **B** Species with the greatest perceived priority in antimicrobial resistance. TB, tuberculosis; NTM, nontuberculous mycobacterium; COPD, chronic obstructive pulmonary disease. *P < 0.05; **P < 0.01; ***P < 0.001

Overall, the three disease areas within chronic lung disease with the greatest perceived AMR burden by the respondents were bronchiectasis (72.8%, 203/279), intensive care (60.6%, 169/279) and mycobacteria (53.0%, 148/279). There were geographical differences in the perceived burden of AMR with *mycobacteria* more frequently picked by respondents in Africa (76.2%, 32/42) compared with Asia (46.0%, 29/63; *p* = 0.002) and Europe (52.2%, 83/159; *p* = 0.005) (Fig. 3A). In contrast, cystic fibrosis (CF) was more frequently identified as having a high AMR burden from respondents in Europe (54.1%, 86/159) compared with Africa (11.9%, 5/42; *P* < 0.001) and Asia (25.4%, 16/63; *P* < 0.001). Bronchiectasis was perceived as having a higher AMR burden in respondents in Asia (87.3%, 55/63) compared with Africa (59.5%, 25/42; *p* = 0.001) and Europe (71.1%, 113/159; 0.01).

Overall, the three species with the greatest perceived AMR burden by respondents were *Pseudomonas aeruginosa* (90.0%, 251/279), *Klebsiella pneumoniae* (52.7%, 147/279) and *Acinetobacter baumannii* (38.0%, 106/279). *Pseudomonas aeruginosa* was picked more frequently as a species with higher AMR burden by respondents in Europe (95.0%, 151/159) and Asia (92.1%, 58/63) compared with Africa (66.7%, 28/42; *P* < 0.001 for both) (Fig. 3B), whereas *Mycobacterium tuberculosis* was picked more frequently by respondents in Africa (71.4%, 30/42) compared with Asia (33.3%, 21/62; *p* < 0.001) and Europe (27.7%, 44/159; *P* < 0.001). By comparison, nontuberculous mycobacteria was picked more frequently by respondents in Europe (37.7%, 60/159) compared with Africa (11.9%, 5/42; *p* = 0.006) and Asia (7.9%, 5/63; *p* < 0.001).

69.2% (193/279) and 62.4% (174/279) of respondents strongly agreed that prior antimicrobial use and inappropriate and/or empirical antimicrobial use respectively were important factors in the acquisition of AMR in chronic respiratory disease (Figure S4). By comparison, only 16.8% (47/279) and 17.6% (49/279) of respondents strongly agreed that person-to-person transmission within lung disease and one-health environmental acquisition of drug-resistant organisms respectively were important factors in AMR acquisition in chronic respiratory disease.

**Fig. 4:**
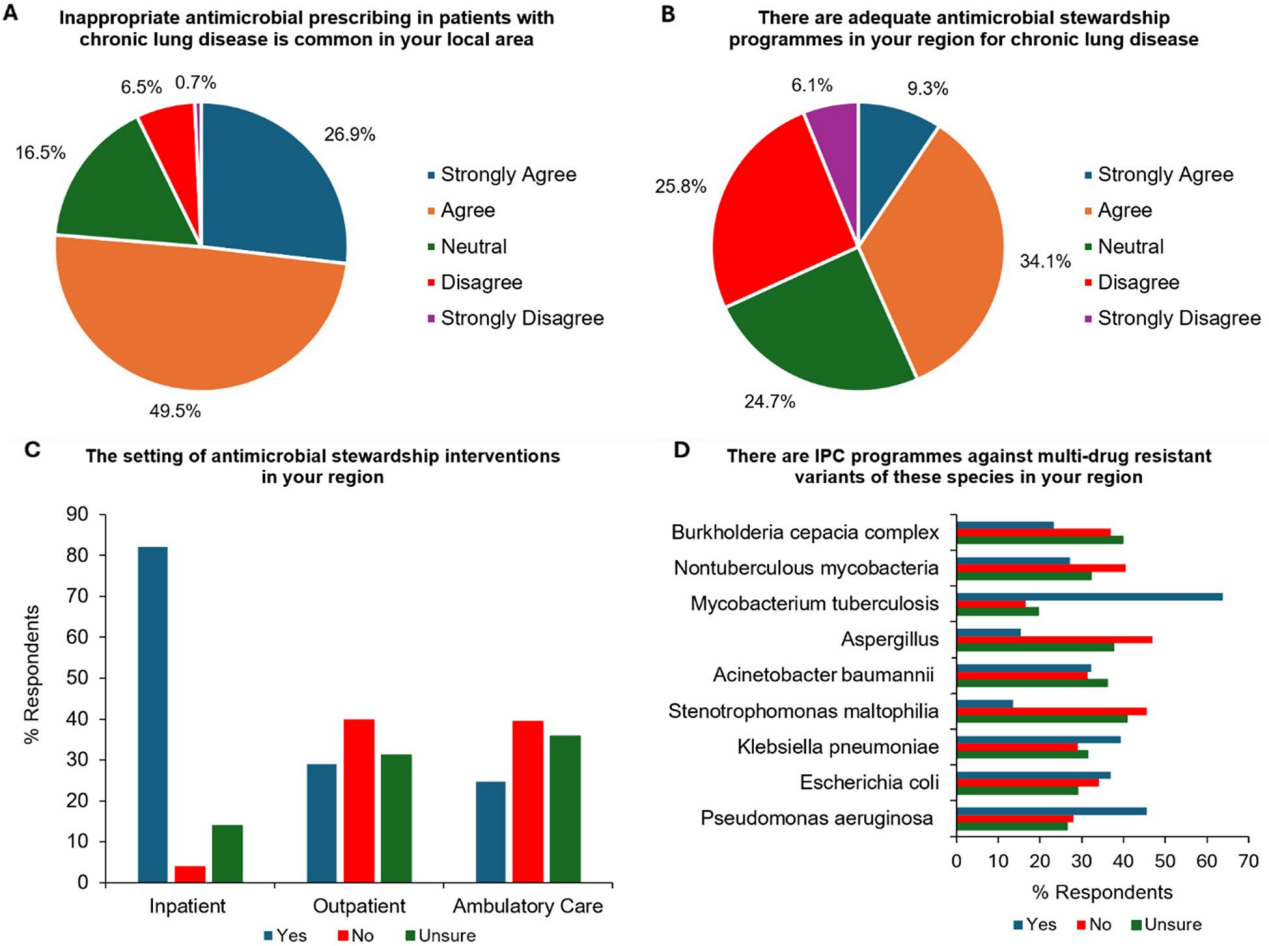
Perceived application of regional antimicrobial stewardship in chronic lung disease. **A** Views on inappropriate antimicrobial prescribing regionally. **B** Views on the quality of regional antimicrobial stewardship programmes. **C** Setting of regional antimicrobial stewardship programmes for chronic respiratory disease. **D** Regional availability of infection prevention and control (IPC) programmes for multi-drug resistant variants of species in chronic lung disease

76.4% (213/279) of respondents agreed or strongly agreed that inappropriate antimicrobial prescribing in chronic respiratory disease is common in their local area (Fig. 4A), while only 43.4% (121/279) agreed or strongly agreed that there are adequate antimicrobial stewardship programmes for chronic respiratory diseases in their regions (Fig. 4B). 82.1% (229/279) stated that antimicrobial stewardship interventions occur at an inpatient setting locally, compared with only 29.0% (81/279) for outpatient and 24.7% (69/279) for ambulatory care settings (Fig. 4C). There were varied levels of specific infection prevention and control (IPC) programmes against MDR variants of common species, with 63.8% (178/279) of respondents indicating the presence of IPC programmes for MDR variants of *Mycobacterium tuberculosis* compared with only 13.6% (38/279) for *Stenotrophomonas maltophilia* (Fig. 4D). When asked to detail the composition of their regional antimicrobial stewardship programmes, the three most common members were infectious disease physicians (61.6%, 172/279), microbiologists (59.1%, 165/279) and respiratory physicians (42.7%, 119/279) (Figure S5).

The three priorities most commonly picked by respondents to improve regional outcomes of antimicrobial-resistant infections in chronic respiratory disease were: better understanding of the epidemiology of AMR strains (55.2%, 154/279), improving healthcare policy and practices (47.3%, 132/279) and better diagnostics (46.2%, 129/279) (Figure S6).

**Fig. 5:**
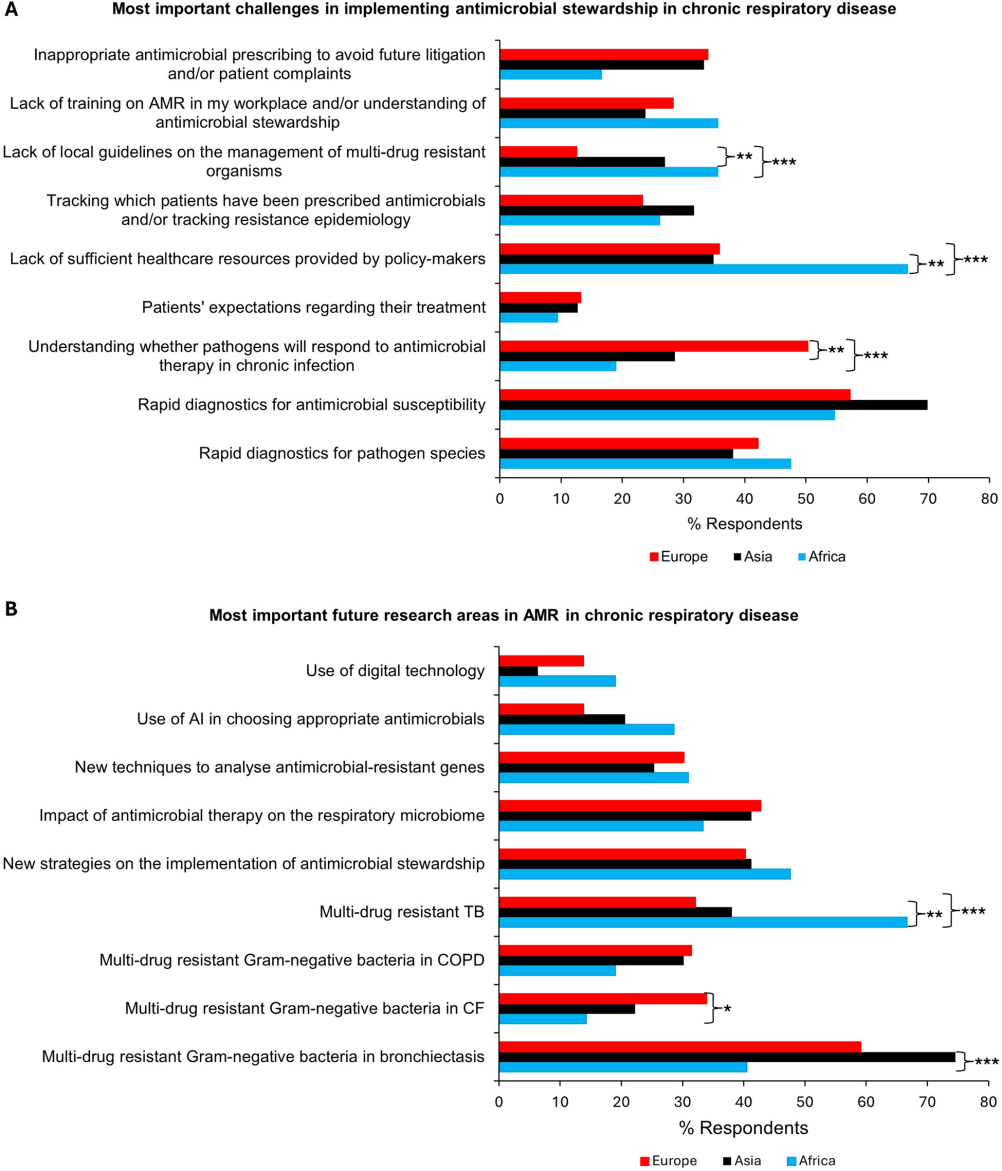
Future outlook of antimicrobial resistance (AMR) in chronic respiratory disease. **A** Views on the most important challenges in the implementation of antimicrobial stewardship in chronic respiratory disease. **B** Views on the most important research priorities in AMR in chronic respiratory disease. AI, artificial intelligence; TB, tuberculosis; COPD, chronic obstructive pulmonary disease; CF, cystic fibrosis. *P < 0.05; **P < 0.01; ***P < 0.001

Overall, the three challenges most frequently picked in implementing antimicrobial stewardship in chronic respiratory disease were developing rapid diagnostics for antimicrobial susceptibility (59.5%, 166/279) and pathogen species (42.7%, 119/279), and understanding whether pathogens will respond to antimicrobials in chronic infection (40.9%, 114/279). More respondents in Africa stated that the lack of healthcare resources provided by policy-makers was a challenge (66.7%, 28/42) compared with Asia (34.9%, 22/62; *p* = 0.001) and Europe (35.8%, 57/159; *P* < 0.001) (Fig. 5A). A lack of local guidelines on the management of MDR species was more commonly identified by respondents in Africa (35.7%, 15/42) and Asia (27.0%, 17/63) compared with Europe (12.6%, 20/159; *P* < 0.001 and *p* = 0.003 respectively).

The top three research priorities in AMR in chronic respiratory disease identified by respondents were MDR Gram-negative bacteria in bronchiectasis (60.6%, 169/279), new strategies on the implementation of antimicrobial stewardship (40.9%, 114/279) and the impact of antimicrobials on the respiratory microbiome (40.5%, 113/279). MDR-tuberculosis (MDR-TB) was a more common research priority amongst healthcare workers in Africa (66.7%, 28/42) compared with Asia (38.1%, 24/63; *p* = 0.004) and Europe (32.1%, 51/159; *p* < 0.001) (Fig. 5B). By comparison, MDR Gram-negative bacteria in CF was a more common research priority in respondents from Europe (34.0%, 54/159) compared with Africa (14.3%, 6/42; *p* = 0.01).

## Discussion

We detail for the first time global views of healthcare workers on the burden and impact of AMR in chronic respiratory disease. Our survey results indicate AMR is perceived to be highly prevalent globally in chronic respiratory disease with significant burden, but with geographical differences in species and diseases of concern, including important differences in perceived high-priority areas in Asia compared to other continents. Our survey results also highlight inconsistencies in local antimicrobial stewardship and IPC programmes and the need for improved health policy and understanding of regional AMR epidemiology within chronic respiratory disease.

Over half of the respondents in our survey reported encountering MDR pathogens daily or weekly in chronic respiratory disease, and over 40% reported that AMR limits their treatment options weekly, indicating a high global AMR burden in chronic respiratory disease. This is corroborated by previous research which has highlighted the prevalence of AMR in chronic respiratory disease, such as a recent systematic review of AMR in COPD where 53% of the included studies reported a resistance rate of > 50% for *Pseudomonas aeruginosa* and 46% for *Streptococcus pneumoniae* [7]. Furthermore, MDR-TB has been shown to cause approximately 13% of all deaths attributed to AMR globally [1, 17]. Further studies have additionally highlighted the importance of MDR pathogens in bronchiectasis, which are associated with worse clinical outcomes and increased mortality [8, 18]. This is evident in our survey where Gram-negative pathogens in bronchiectasis were perceived as a key research priority area, especially by respondents from Asia [19]. A limitation of our survey is the likely bias towards the inclusion of healthcare professionals with an interest in AMR. Nevertheless, given the limited epidemiological data on the true global AMR burden in chronic respiratory disease, our survey highlights the perceived significant burden and the need for further research to corroborate findings.

There were expected geographical variations in the conditions and species with greatest perceived AMR burden, such as TB predominating in Africa and CF in Europe [20, 21]. Bronchiectasis and research into Gram-negative pathogens affecting bronchiectasis were considered high priorities more frequently by respondents in Asia, perhaps reflecting the increasing prevalence recently reported in this region [22]. The establishment of international bronchiectasis research networks including countries in Asia has led to better understanding of geographical differences in aetiology and microbiology, and provides a template to better understand specific regional AMR outcomes and challenges in chronic lung disease [23]. Our survey results also highlight the AMR burden in species such as *Klebsiella pneumoniae* and *Acinetobacter baumannii,* which are comparatively less well studied in the context of chronic lung disease. *Klebsiella pneumoniae* had one of the greatest perceived AMR burdens by our survey respondents and has been shown to be independently associated with bronchiectasis and COPD exacerbation mortality [23, 24]. There is additionally limited literature on the role of *Acinetobacter baumannii* in chronic respiratory disease, despite it being regarded particularly important by our survey respondents, especially from Asia, where prior studies have identified it as a common MDR bacterial species within bronchiectasis [18]. To date, there is little understanding of the drivers and transmission dynamics within airway colonisation of chronic lung disease and whether repeated antibiotic use in these patients contributes to the high prevalence of MDR and hypervirulent *Klebsiella* and *Acinetobacter* strains, isolated from community- and hospital-acquired pneumonia in the Asian subcontinent. In our survey, IPC programmes for *Klebsiella pneumoniae* and *Acinetobacter baumannii* were reported to be in place by less than 50% of respondents in their institutions and less commonly than other species, such as *Pseudomonas aeruginosa*. Further research is urgently needed to better understand transmission dynamics and whether IPC programmes for these pathogens could translate to improved regional AMR outcomes.

Our survey results additionally show that lack of appropriate antimicrobial stewardship in chronic respiratory disease is perceived to be a global problem and challenge. 76.2% of respondents reported that inappropriate antimicrobial prescribing is common in their area. This corroborates recent research which revealed that within analysis of over 26,000 patient visits in a primary care system, 69.0% of antibiotic prescriptions for respiratory infections were inappropriate [25], with similar within exacerbations of airways disease in 16–58% of patients [26–28]. Inappropriate antimicrobial prescribing was also perceived by survey respondents as one of the most important factors in the acquisition of AMR in chronic respiratory disease. Given the drive towards outpatient and ambulatory care, including management of exacerbations (such as parenteral antibiotic therapy in bronchiectasis) within chronic respiratory disease, our survey reveals limited implementation of antimicrobial stewardship in these settings, despite evidence that outpatient antimicrobial stewardship programmes can lead to improved adherence to guidelines for respiratory tract infections [29, 30]. However, recent global modelling studies examining socioeconomic, anthropogenic and environmental indicators, alongside country-level rates of AMR in humans and food-producing animals, suggest that limiting antimicrobial prescription alone is unlikely to be sufficient to combat global AMR prevalence [31, 32]. Whether targeted antimicrobial stewardship in high-risk chronic lung disease settings can limit AMR prevalence and improve outcomes is unclear and requires further prospective research.

There remain significant hurdles to be overcome in the implementation of antimicrobial stewardship in chronic respiratory disease. As highlighted by our survey respondents, the availability and use of rapid diagnostics for AMR and pathogen species detection is an ongoing challenge. Although novel technologies are being developed, there is a requirement for prospective trials to determine the effectiveness of rapid diagnostics, such as novel sequencing or multiplex rapid detection systems, to enable decision-making around the need, targeting and cessation of antimicrobial therapies in chronic respiratory disease [33, 34]. Antimicrobial prescribing behaviour is additionally a complex process, influenced by a broad range of determinants, and understanding how to translate evidence of effectiveness of novel diagnostic approaches to exacerbation management in chronic lung disease clinical practice, where empiric treatment is commonplace, will be required.

Further challenges include the presence of polymicrobial infection and understanding the aetiology of exacerbation in the context of chronic microbial colonisation [35]. The advent of microbial sequencing has shown that microbial interaction networks are associated with exacerbation risk (rather than microbial abundance alone), with distinct “resistotypes” (clusters of AMR genes) identified in recent studies correlating with clinical outcomes in chronic respiratory disease, in particular bronchiectasis [8, 36, 37]. Thus, a paradigm shift in antimicrobial prescription may be needed to tackle the complex microbial interplay present in chronic lung disease exacerbations. A lack of healthcare resources was a more prevalent challenge for respondents in Africa compared to the other continents, reflecting the general trend of lower healthcare expenditure per capita in African countries [38]. Given the global spread of AMR, international collaboration is thus imperative to facilitate improved resource allocation in low-middle income countries to combat the global spread and burden of AMR.

## Conclusions

Our survey for the first time presents the views of healthcare worker on the impact and burden of AMR in chronic respiratory disease, with AMR being perceived as a significant challenge and threat globally. Whilst daunting challenges remain, it is encouraging that most respondents felt that AMR was a key priority for policymakers within their region. The survey however clearly identifies areas where there is currently a lack of clarity and focus, with limited implementation of antimicrobial stewardship, and unclear evidence and international guidance around requirements for IPC programmes for many MDR pathogens in chronic respiratory disease. Further research is needed to better understand infection transmission dynamics of AMR pathogens in chronic respiratory disease, as well as the development of publicly accessible real-time AMR surveillance systems at regional levels to guide antimicrobial stewardship and prescribing, which was identified as the highest priority to improve current outcomes. Our survey indicates a need for international collaboration to urgently facilitate a prospective rigorous approach to accurately understand AMR epidemiology and burden in chronic respiratory disease.

## Supplementary Information

Additional file 1: Figure S1 Background details for survey respondentsArea of work.Patient population managed.Disease area primarily managed.How often respondents prescribed antimicrobials and/or review antimicrobial prescriptions and/or advise patients regarding antimicrobial use.

Additional file 2: Figure S2 Frequency with which respondents see patients die due to a lack of treatment options as a result of antimicrobial resistance.

Additional file 3: Figure S3 Respondents’ views on the importance of various factors in the acquisition of antimicrobial resistancein chronic lung disease.

Additional file 4: Figure S4 Composition of the respondents’ regional antimicrobial stewardship teams.

Additional file 5: Figure S5 Priorities for improving regional outcomes of antimicrobial-resistantinfections in chronic respiratory disease.

Additional file 6: Table S1 The online survey sent to healthcare workers with an interest in antimicrobial resistance in respiratory disease.

Additional file 7.

## Data Availability

The data that support the findings of this study are available from the corresponding author upon reasonable request.

## Abbreviations

AMR: Antimicrobial resistance
ERS: European Respiratory Society
MDR: Multi-drug resistant
CF: Cystic fibrosis
IPC: Infection prevention and control
MDR-TB: Multi-drug resistant tuberculosis
COPD: Chronic obstructive pulmonary disease
