# Supplementary figures and images for: Healthcare worker views on antimicrobial resistance in chronic respiratory disease
Source: Antimicrob Resist Infect Control. 2025 Jan 22;14:1. doi: 10.1186/s13756-025-01515-8 (PMC11752958; doi:10.1186/s13756-025-01515-8)

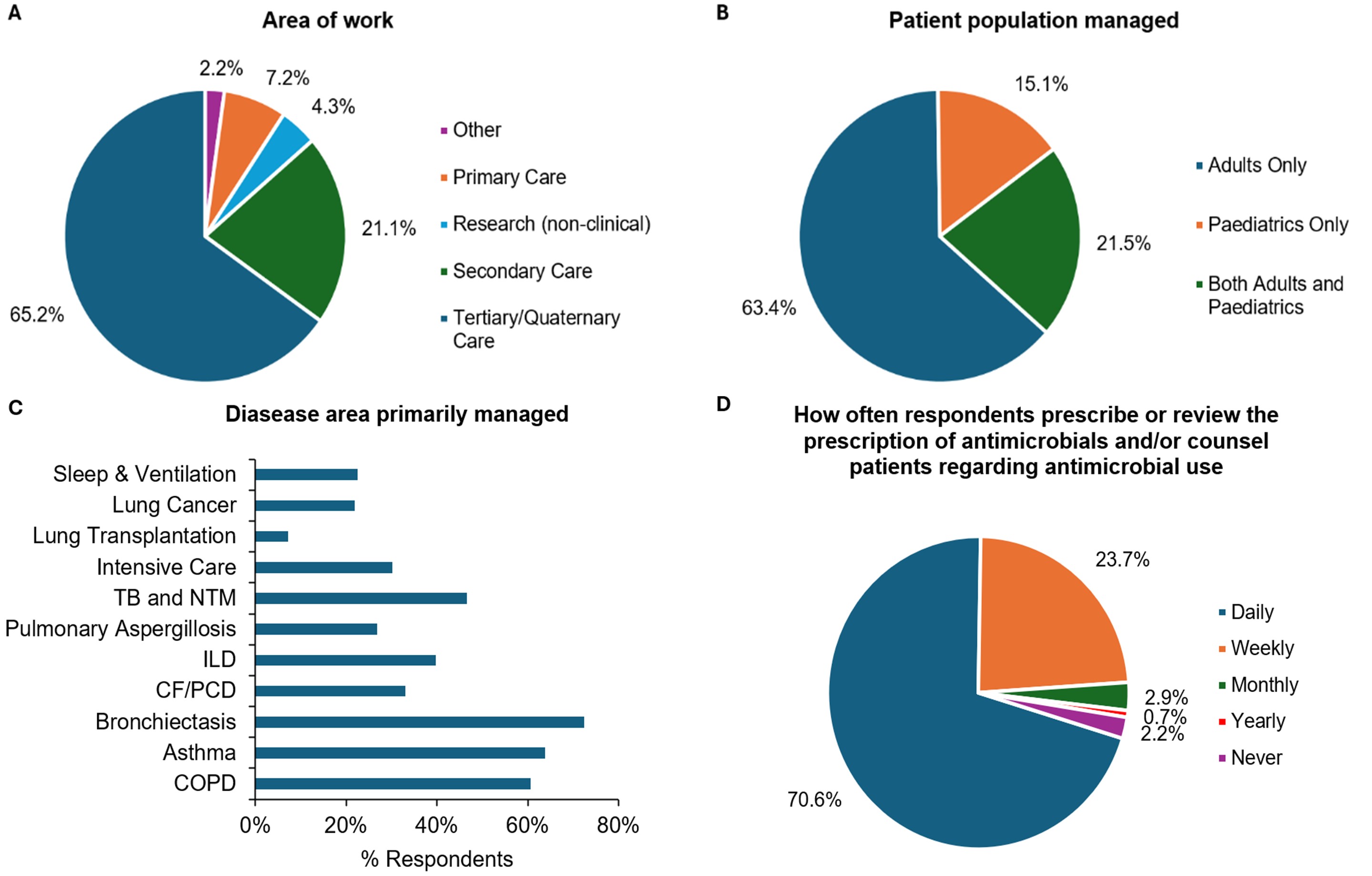

Supplement: Supplementary file 1 — Additional file 1: Figure S1 Background details for survey respondentsArea of work.Patient population managed.Disease area primarily managed.How often respondents prescribed antimicrobials and/or review antimicrobial prescriptions and/or advise patients regarding antimicrobial use. [file 13756_2025_1515_MOESM1_ESM.jpg]

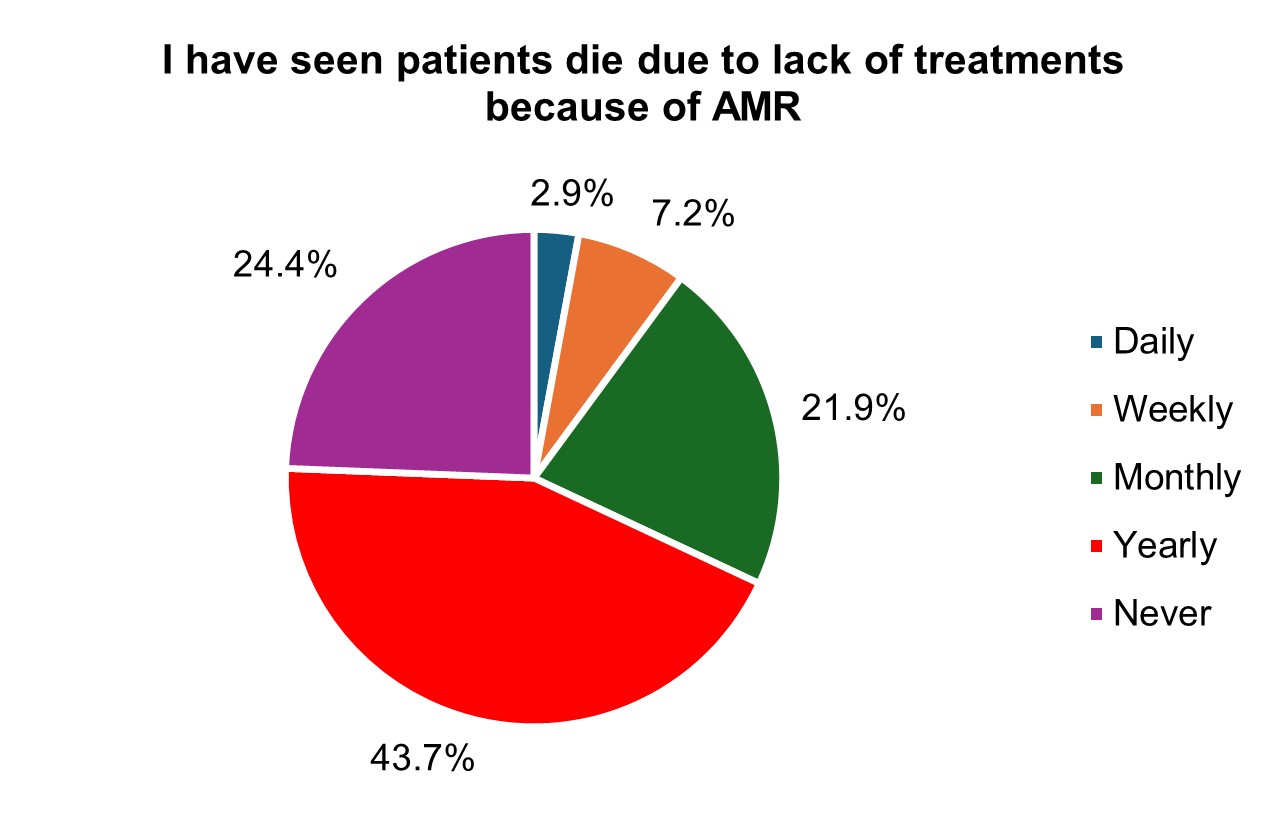

Supplement: Supplementary file 2 — Additional file 2: Figure S2 Frequency with which respondents see patients die due to a lack of treatment options as a result of antimicrobial resistance. [file 13756_2025_1515_MOESM2_ESM.jpg]

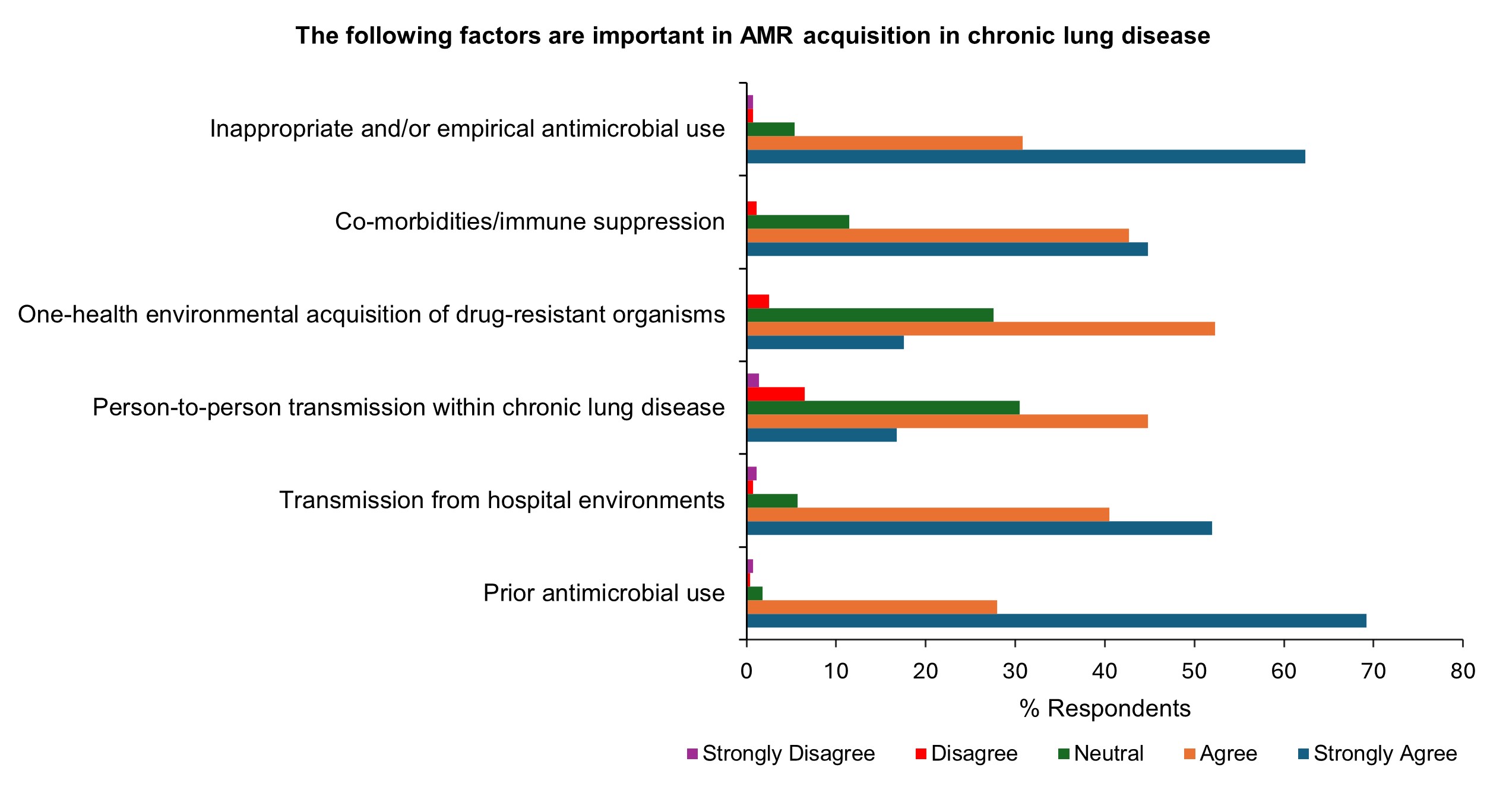

Supplement: Supplementary file 3 — Additional file 3: Figure S3 Respondents’ views on the importance of various factors in the acquisition of antimicrobial resistancein chronic lung disease. [file 13756_2025_1515_MOESM3_ESM.jpg]

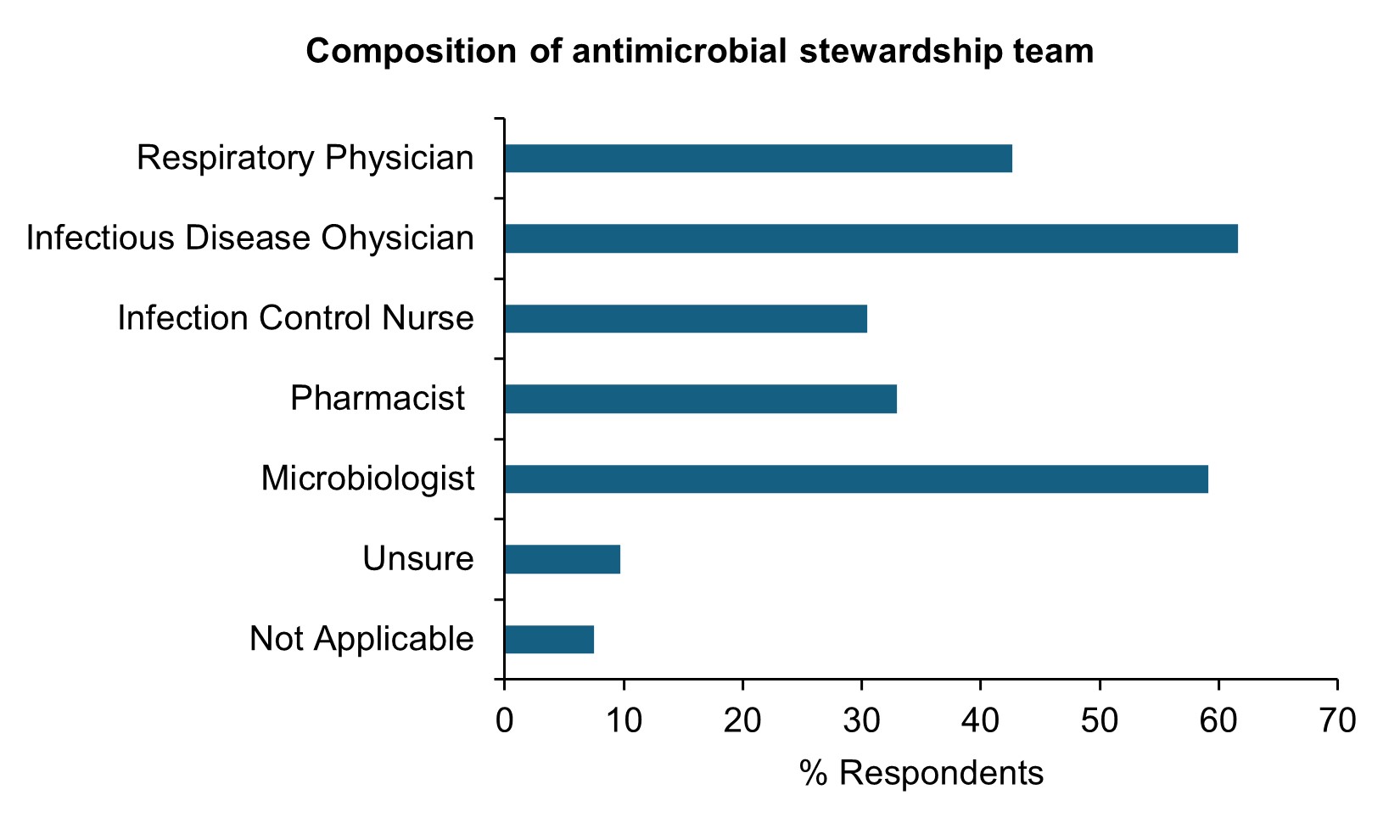

Supplement: Supplementary file 4 — Additional file 4: Figure S4 Composition of the respondents’ regional antimicrobial stewardship teams. [file 13756_2025_1515_MOESM4_ESM.jpg]

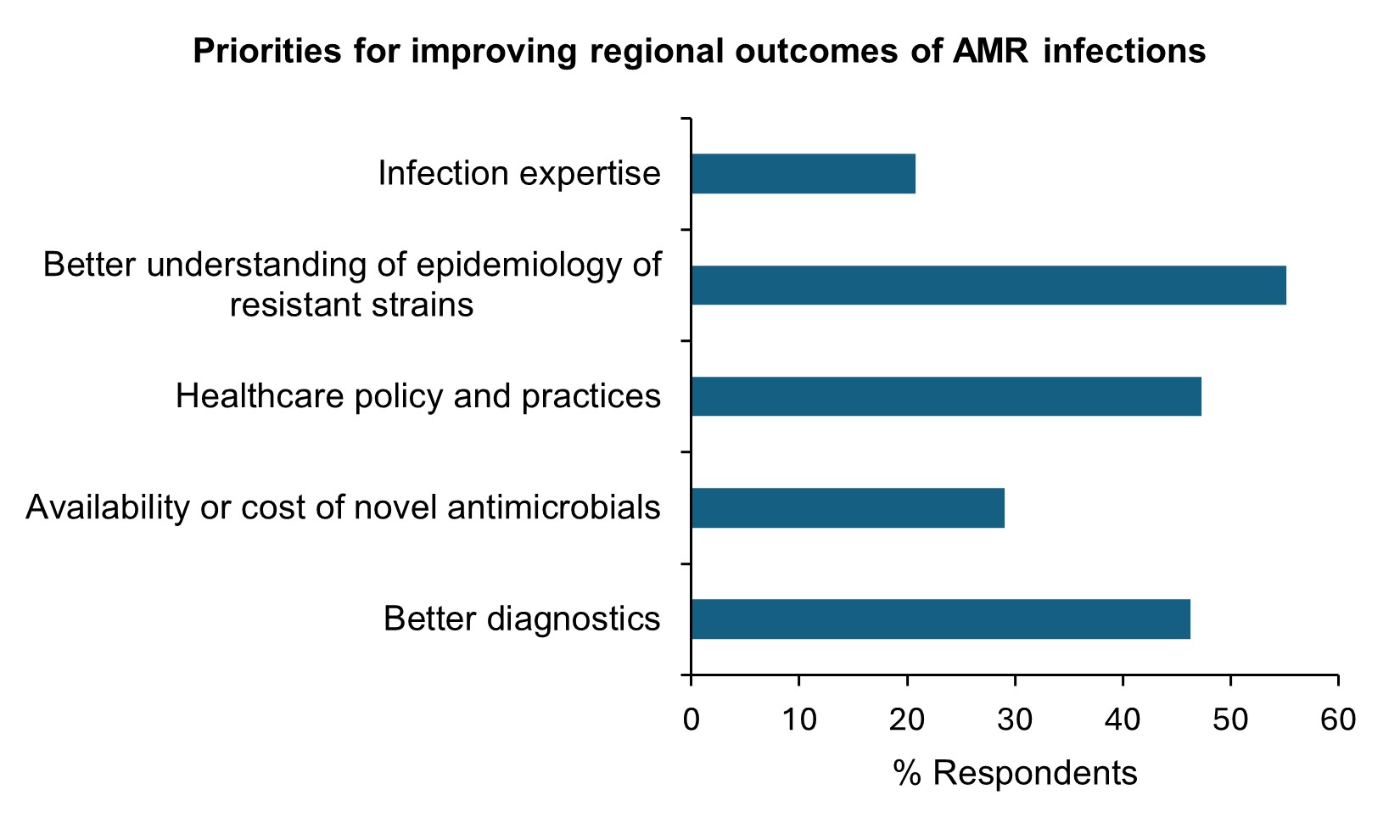

Supplement: Supplementary file 5 — Additional file 5: Figure S5 Priorities for improving regional outcomes of antimicrobial-resistantinfections in chronic respiratory disease. [file 13756_2025_1515_MOESM5_ESM.jpg]
