# Supplementary material for: Healthcare worker views on antimicrobial resistance in chronic respiratory disease
Source: Antimicrob Resist Infect Control. 2025 Jan 22;14:1. doi: 10.1186/s13756-025-01515-8 (PMC11752958; doi:10.1186/s13756-025-01515-8)
Supplement: Supplementary file 6 — Additional file 6: Table S1 The online survey sent to healthcare workers with an interest in antimicrobial resistance in respiratory disease. [file 13756_2025_1515_MOESM6_ESM.docx]

| **Demographics** | - Country of practice - How long have you been practising? Specify in years - Gender   - Male   - Female   - Non-binary   - Other   - Prefer not to say - Age range   - 18-24   - 25-34   - 35-44   - 45-54   - 55-64   - 65-74   - 75+ - Profession   - Clinician   - Nurse   - Pharmacist   - Physiotherapist/occupational therapist   - Laboratory Scientist (non-clinician)   - Other **[Free text box to specify]** - Area of work   - Primary care   - Secondary care   - Tertiary/quaternary care   - Research institute (no clinical work)   - Other **[Free text box to specify]** - Do you look after:   - Adult patients   - Paediatric patients   - Both adults and paediatric patients - How often do you prescribe, review the prescription and/or provide advice regarding antibiotics use to patients?   - Daily   - Weekly   - Monthly   - Yearly   - Never - What disease area do you most commonly work in (you can pick more than one)?   - COPD   - Asthma   - Bronchiectasis   - Cystic fibrosis   - ILD   - Pulmonary aspergillosis   - TB and NTM   - Intensive care   - Lung transplant   - Lung cancer   - Sleep & ventilation   - Other **[Free text box to specify]** |
| --- | --- |
| **Antimicrobial Resistance** | - I encounter multi-drug resistant organisms in respiratory infections   - Daily   - Weekly   - Monthly   - Yearly   - Never - Antimicrobial resistance limits my treatment options for respiratory infections   - Daily   - Weekly   - Monthly   - Yearly   - Never - I have seen patients with respiratory infections clinically deteriorate due to the lack of treatment options as a result of antimicrobial resistance   - Daily   - Weekly   - Monthly   - Yearly   - Never - I have seen patients die due to the lack of treatment options as a result of antimicrobial resistance   - Daily   - Weekly   - Monthly   - Yearly   - Never - Antimicrobial resistance in chronic lung disease is considered an important topic by policymakers in my region/district   - Strongly agree   - Agree   - Neutral   - Disagree   - Strongly disagree - Pick 3 disease areas where you feel that antimicrobial resistance has the largest current burden in your region/practice - COPD - Asthma - Bronchiectasis - Cystic fibrosis - ILD - Pulmonary aspergillosis - TB and NTM - Intensive care - Lung transplant - Lung cancer - Sleep & ventilation - Pick 3 species which are the highest priority in the field of antimicrobial resistance in chronic lung disease   - Pseudomonas aeruginosa - Escherichia coli (E. coli) - Klebsiella pneumoniae - Stenotrophomonas maltophilia - Acinetobacter baumannii - Mycobacterium tuberculosis - Aspergillus - Nontuberculous mycobacteria species - Burkholderia cepacia complex - Other **[Free text box to specify]** - The following factors are important in antimicrobial resistance acquisition in chronic lung disease:  \|  \| Strongly Agree \| Agree \| Neutral \| Disagree \| Strongly Disagree \| \| --- \| --- \| --- \| --- \| --- \| --- \| \| Prior antimicrobial use \|  \|  \|  \|  \|  \| \| Transmission from hospital environments \|  \|  \|  \|  \|  \| \| Person-to-person transmission within chronic lung disease \|  \|  \|  \|  \|  \| \| One-health environmental acquisition of drug-resistant organisms \|  \|  \|  \|  \|  \| \| Co-morbidities/immune suppression \|  \|  \|  \|  \|  \| \| Inappropriate and/or empirical antimicrobial use \|  \|  \|  \|  \|  \|  - Pick 2 areas which are priorities for improving your regional outcomes of antimicrobial-resistant infections in chronic lung diseases: - Better diagnostics - Availability or cost of novel antimicrobial therapy - Healthcare policy/practices - Better understanding of regional/national epidemiology - Infection expertise - Other **[Free text box to specify]** |
| **Antimicrobial Stewardship** | - Inappropriate antimicrobial prescribing in patients with chronic lung disease is common in my local area (including local hospital)   - Strongly agree   - Agree   - Neutral   - Disagree   - Strongly disagree - There are adequate antimicrobial stewardship programmes in your region/district (including our facility) for individuals with chronic lung disease   - Strongly agree   - Agree   - Neutral   - Disagree   - Strongly disagree - If you have antimicrobial stewardship programmes in your region/district, who is part of this: - Respiratory physicians - Infection Disease physicians - Microbiologists - Pharmacists - Infection control nurses - Not applicable (there are no antimicrobial stewardship programmes in my region) - Other **[Free text box to specify]** - Where do antimicrobial stewardship interventions take place in your region/district?  \|  \| Yes \| No \| Not sure \| \| --- \| --- \| --- \| --- \| \| Inpatient setting \|  \|  \|  \| \| Outpatient setting \|  \|  \|  \| \| Ambulatory Care setting \|  \|  \|  \|      - Pick the 3 most important challenges in implementing antimicrobial stewardship in chronic respiratory disease - Rapid diagnostics for pathogen species - Rapid diagnostics for antimicrobial susceptibility - Understanding whether pathogens will respond to antimicrobial therapy in chronic infection - Patients’ expectations regarding their treatment - Lack of sufficient healthcare resources provided by policy-makers e.g. limited microbiological facilities, lack of funding for antimicrobial stewardship programmes - Tracking which patients have been prescribed antimicrobials and/or tracking resistance epidemiology - Lack of guidelines in my centre regarding the management of multi-drug resistant organisms - Lack of training on antimicrobial resistance at my workplace and/or understanding of antimicrobial stewardship - Inappropriate antimicrobial prescribing by clinicians to avoid future litigation/patient complaints - Other **[Free text box to specify]** - There are infection prevention and control (IPC) programmes specifically against *multi-drug resistant variants* of the following species in my district/region  \|  \| Yes \| No \| Not sure \| \| --- \| --- \| --- \| --- \| \| Pseudomonas aeruginosa \|  \|  \|  \| \| Escherichia coli (E. coli) \|  \|  \|  \| \| Klebsiella pneumoniae \|  \|  \|  \| \| Stenotrophomonas maltophilia \|  \|  \|  \| \| Acinetobacter baumannii \|  \|  \|  \| \| Mycobacterium tuberculosis \|  \|  \|  \| \| Nontuberculous mycobacteria species \|  \|  \|  \| \| Burkholderia cepacia complex \|  \|  \|  \|  - Antimicrobial susceptibility patterns influence my choice of:  \|  \| Strongly Agree \| Agree \| Neutral \| Disagree \| Strongly Disagree \| \| --- \| --- \| --- \| --- \| --- \| --- \| \| Oral antimicrobials \|  \|  \|  \|  \|  \| \| Intravenous (IV) antimicrobials \|  \|  \|  \|  \|  \| \| Nebulised antimicrobials \|  \|  \|  \|  \|  \| |
| **Future Outlook** | - Pick the 3 most important areas that require further research - Multi-drug resistant Gram-negative bacteria in bronchiectasis - Multi-drug resistant Gram-negative bacteria in cystic fibrosis - Multi-drug resistant Gram-negative bacteria in COPD - Multi-drug resistant TB - New strategies on the implementation of antimicrobial stewardship - Impact of antimicrobial therapy on the respiratory microbiome - New techniques to analyse antimicrobial-resistant genes - Use of artificial intelligence in choosing appropriate antimicrobials - Use of digital technology, including tele-medicine and/or virtual wards in the management of outpatients with multi-drug resistant respiratory infections - Other **[Free text box to specify]** |

**Table S1** The online survey sent to healthcare workers with an interest in antimicrobial resistance in respiratory disease.
