# Supplementary material for: Healthcare worker views on antimicrobial resistance in chronic respiratory disease
Source: Antimicrob Resist Infect Control. 2025 Jan 22;14:1. doi: 10.1186/s13756-025-01515-8 (PMC11752958; doi:10.1186/s13756-025-01515-8)
Supplement: Supplementary file 7 — Additional file 7. [file 13756_2025_1515_MOESM7_ESM.docx]

| **Occupation** | **Number (%)** |
| --- | --- |
| Clinician | 255 (91.4) |
| Scientist | 8 (2.9) |
| Physiotherapist | 7 (2.5) |
| Pharmacist | 4 (1.4) |
| Nurse | 2 (0.7) |
| Other (unspecified) | 3 (1.1) |

**Table S2** Occupations of the respondents to the online survey
